# Thrombin-Binding Aptamer with Inversion of Polarity Sites (IPS): Effect on DNAzyme Activity and Anticoagulant Properties

**DOI:** 10.3390/ijms22157902

**Published:** 2021-07-23

**Authors:** Joanna Kosman, Bernard Juskowiak

**Affiliations:** Department of Bioanalytical Chemistry, Faculty of Chemistry, Adam Mickiewicz University, Uniwersytetu Poznanskiego 8, 61-614 Poznan, Poland; juskowia@amu.edu.pl

**Keywords:** G-quadruplex, DNAzyme, inversion of polarity, thrombin, TBA

## Abstract

In this work we examined the properties of thrombin-binding aptamer (TBA) modified by the introduction of inversion of polarity sites (IPS) in order to assess the effect of modification on the activation of TBA to serve as DNAzyme with peroxidase-like activity. Two oligonucleotides were designed to possess one (IPS1) or three (IPS2) inversion sites. TBA typically forms antiparallel G-quadruplexes with two G-tetrads, which exhibits very low DNAzyme peroxidise activity. DNAzyme activity is generally attributed to parallel G-quadruplexes. Hence, inversion of polarity was introduced in the TBA molecule to force the change of G-quadruplex topology. All oligonucleotides were characterized using circular dichroism and UV-Vis melting profiles. Next, the activity of the DNAzymes formed by studied oligonucleotides and hemin was investigated. The enhancement of peroxidase activity was observed when inversion of polarity was introduced. DNAzyme based on IPS2 showed the highest peroxidase activity in the presence of K^+^ or NH_4_^+^ ions. This proves that inversion of polarity can be used to convert a low-activity DNAzyme into a DNAzyme with high activity. Since TBA is known for its anticoagulant properties, the relevant experiments with IPS1 and IPS2 oligonucleotides were performed. Both IPS1 and IPS2 retain some anticoagulant activity in comparison to TBA in the reaction with fibrinogen. Additionally, the introduction of inversion of polarity makes these oligonucleotides more resistant to nucleases.

## 1. Introduction

Thrombin is a multifunctional serine protease that plays a key role in thrombosis, homeostasis, and inflammation. In the physiological homeostatic process, fibrin clot itself exhibits significant thrombin-binding potential for the concentration of free thrombin in blood plasma decreases due to its binding not only to fibrinopeptide A/B cleavage sites on the fibrinogen molecule, but also by binding to fibrinogen through an anion binding site (exosite 1) [1]. Thrombin-binding aptamers are thus important in the medical and clinical fields. Aptamers, nucleic acid equivalents of antibodies, are single stranded DNA or RNA molecules that can selectively bind with other molecules [2]. They are selected through the SELEX procedure (Systematic Evolution of Ligands by EXponential enrichment) and each year new aptamers for biologically significant molecules are developed e.g., adenosine, vascular endothelial growth factor or cancer cells [3,4,5]. Several aptamers are studied for their therapeutic application in various stages of clinical trials. The interest in aptamers results from many advantages over protein antibodies, such as simple chemical synthesis, simple modification, small size and low variability between batches [6]. One of the most studied aptamer is the thrombin-binding aptamer (TBA). It was proven that this aptamer forms a secondary structure of the G-quadruplex [7]. The G-quadruplex (G4 DNA) is an advanced structure formed by single-stranded nucleic acid stabilized by Hoogsteen hydrogen bonds, electrostatic interactions, and hydrophobic stacking between guanosine residues [8]. TBA was proven to possess anticoagulant properties and has been clinically tested [9]. Moreover, the antiproliferative activity of this aptamer was also reported [10]. Taking into account the biological significance of this aptamer, a lot of research has been undertaken to enhance its anticoagulant and antiproliferative activities. These were achieved by chemical modification of the bases or sugar-phosphate backbone of the DNA molecule. The studied modifications included among others: locked nucleic acid residues [11], 2′-OMe-RNA residues [12], unlocked nucleic acid residues [13], d- and l-isothymidine analogues [14], and cyclic TBA analogue [15].

The other TBA modification that attracted a lot of attention is the inversion of DNA polarity. Martino et al. first studied TBA oligonucleotide containing a single 5′-5′ inversion of polarity site [16]. They proved that this oligonucleotide formed a chair-type G-quadruplex with two G-tetrads similarly as the parent TBA molecule. The same group synthesized a mini-library of TBA analogues containing inversion of polarity sites [17]. The X-ray analysis of complex between modified TBA and thrombin was also undertaken [18,19]. However, the conformation of modified TBA molecules or details on interaction with thrombin were not solved. Later research on TBA analogues with inversion of polarity sites focused on the introduction of two inversion sites in order to enhance its thermal stability and thrombin-binding affinity [20]. Moreover, beside inversion of polarity, L-residues were introduced to further study the effect of modification on aptamer structures [21,22]. It was proven that such analogues lost anticoagulant activity but gained antiproliferative activity against cancer lines [22,23]. It was also discovered that the addition of extra-residues at the end of the aptamer linked through 3′-3′ or 5′-5′ phosphodiester bonds resulted in higher thermal stability [24]. The other application of TBA analogues was proposed by Zhou et al., who used these analogues for recognition of isoquinoline alkaloids [25].

Interestingly, studies on G-quadruplexes with inversion of polarity sites did not limit to TBA oligonucleotides. The studies focused not only on other DNA sequences, but also on oligonucleotides capable of forming DNAzymes. G-quadruplexes are able to form complex with hemin molecules and consecutively possess peroxidase-mimicking activity [26]. G-quadruplex/hemin DNAzymes are widely used in bioanalysis thanks to many advantages over protein enzymes (thermal stability, simple synthesis and purification) [27]. Cao and co-workers studied the effect of 3′ and 5′ deoxyadenosine caps on the activity of DNAzyme based on tetramolecular G-quadruplexes [28]. Analogues with inversion of polarity sites were also used for the modification of unimolecular G-quadruplexes, which translated into an enhancement of their catalytic activity [29]. Virgilio et al. examined tetramolecular G-quadruplexes with 5′-5′ internal inversion of polarity sites and double 3′ external G-quartets to obtain thermally stable DNAzymes with enhanced peroxidase activity [30].

**Scheme 1:**
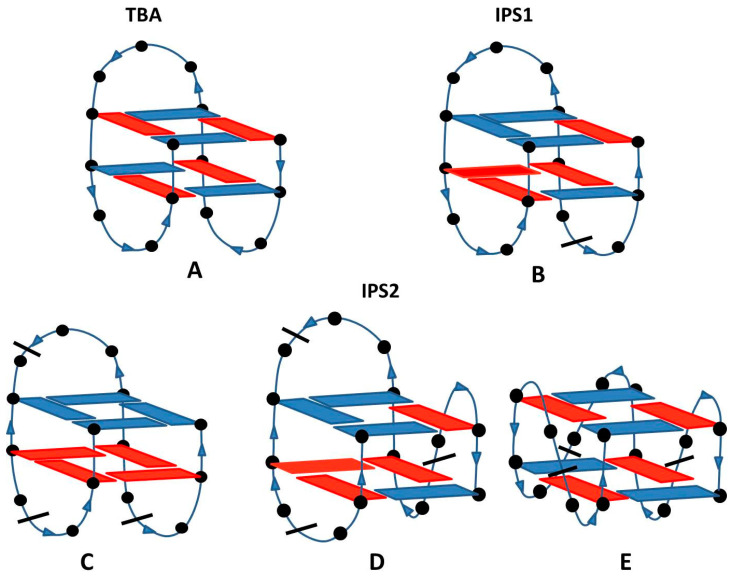
Proposed structures formed by studied oligonucleotides: TBA (**A**), IPS1 (**B**) and IPS2 (**C**,**D** or **E**). Red and blue squares indicate *syn* and *anti* guanosine residues. Inversion of polarity sites are indicated by black line and 5′-3′ orientation is indicated by arrows.

In this work we selected a TBA sequence and designed oligonucleotides with one or three inversion of polarity sites (Scheme 1). TBA was chosen for studying the IPS effect on DNAzyme activity because in the presence of hemin, it does not possess peroxidase activity [31]. It was proven that the highest activity of DNAzymes was observed for parallel G-quadruplexes, which possessed propeller-like loops and exposed terminal tetrads [32]. TBA forms an antiparallel G-quadruplex, in which two guanosines in the G-tetrad possess a *syn*-conformation and two others exhibit an *anti*-conformation of guanosine residues (Scheme 1A). In parallel G-quadruplexes all strands consist of guanosine residues in the same conformation, typically *anti* conformation. We designed a TBA analogue IPS1 with one inversion site of polarity to switch direction of one of the strands to force the formation of 3 + 1 topology (3′-GGT-5′-5′-TGGTGTGGTTGG-3′). Furthermore, to force the direction of all strands in one direction, we introduced three inversion of polarity sites to obtain IPS2 (3′-GGT-5′-5′-TGGTG-3′-3′-TGGT-5′-5′-TGG-3′). We examined the effect of such modifications on the G-quadruplex structure, thermal stability, and catalytic properties, as well as anticoagulant activity.

## 2. Results

**Figure 1:**
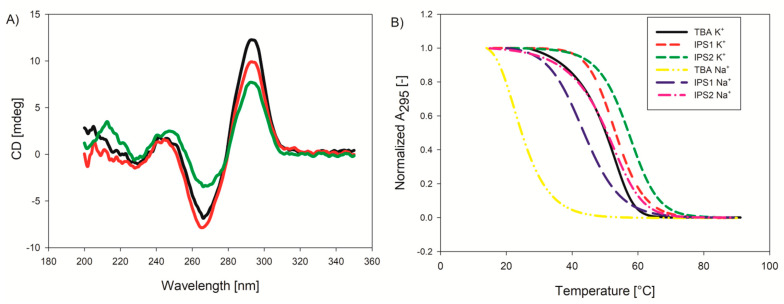
CD spectra and melting profiles of studied oligonucleotides. (**A**) CD spectra: black—TBA, red—IPS1, green—IPS2; (**B**) Melting profiles of studied oligonucleotides in the presence of potassium or sodium cations. Conditions: 10 mM Tris-HCl (pH = 8.0), 100 mM salt (NaCl or KCl), 2 µM DNA.

TBA is known to form an antiparallel G-quadruplex with a chair-type conformation. This unimolecular G-quadruplex consists of two G-tetrads linked by an edge-wise loop and two lateral loops [7]. Circular dichroism (CD) is a technique generally used to differentiate G-quadruplex topologies. Antiparallel G4 topology is characterized by a positive band at approximately 290 nm and a negative band at approximately 260 nm. We used the CD technique to verify whether IPS analogues form G-quadruplex structures. CD spectra should also reveal the effect of the introduction of inversion of polarity sites on the topology of formed G-quadruplexes. Recorded CD spectra for all investigated systems are shown in Figure 1A. TBA oligonucleotide exhibited positive bands at 295 nm and 247 nm and negative band at 266 nm, which is consistent with previous reports [7]. Both IPS1 and IPS2 also showed similar characteristic bands in the CD spectra. IPS1 was previously studied by Esposito et al. and the authors discussed the antiparallel CD spectrum in terms of the 3 + 1 strand arrangement and alternating *syn*-*anti* glycosidic conformation along each strand [16]. The CD spectra of studied oligonucleotides in the presence of sodium and ammonium ions also showed similar characteristics with a positive band at approximately 295 nm and a negative band at approximately 260 nm (Figure S1). Additionally, the CD spectra of studied oligonucleotides were recorded for samples after slow (3 h RT) and fast cooling (15 min on ice). No difference was observed in the spectra suggesting that the same structure was observed under both conditions (Figure S2). It is difficult to determine definitely the topology of G-quadruplexes based only on CD spectra because of the possibility of the formation of multiple topologies. Additionally, TBA is formed by only two G-tetrads and because of it, the spectra could look different than those for more common three-tetrad G-quadruplexes. This is especially apparent for 3 + 1 G-quadruplexes. For a typical 3 + 1 G-quadruplex, like telomeric G-quadruplex in the presence of potassium cation, the glycosidic conformation for strands is *syn*-*syn-anti* or *anti*-*anti-syn*. As a result, both positive bands at approximately 260 and 295 nm are observed. However, in the case of IPS1, only two tetrads are present and because of that, even though the G-quadruplex is a 3 + 1 type, the arrangement in each strand is *syn*-*anti* thus, the CD spectrum resembles that for the antiparallel structure. This was discussed in more detail by Esposito et al. [16,23]. The IPS1 and IPS2 spectra have characteristic bands for antiparallel G-quadruplexes, however, the decrease in the intensity of positive bands at 295 nm and negative bands at 266 nm, with a slight shift of the band at approximately 240 nm toward a higher wavelength for the IPS2 oligonucleotide, show that there are some topological differences between the topologies of IPS1 and IPS2. Inspection of the expected structures of IPS2 with three inversion polarity sites indicates that there are two possible arrangements of a G-quadruplex structure: a 3 + 1 strand arrangement with a single propeller loop (Scheme 1D) or an antiparallel structure of the G-quadruplex with three propeller loops (Scheme 1E). Both structures may explain the antiparallel-like CD spectrum of the IPS2 molecule. The parallel structure (Scheme 1C) should be excluded since such strand arrangement is expected to exhibit a positive CD band at 260 nm [22].

Melting profiles were recorded to determine the thermal stability of studied oligonucleotides (Figure 1B, Table 1). The melting temperature of TBA in the presence of 100 mM KCl was determined to be 51.2 °C and the introduction of inversion of polarity sites resulted in a slight increase in the melting temperature of approximately 3.2 and 7 °C for IPS1 and IPS2, respectively. Higher differences in T_M_ values were observed in the presence of sodium cation. The TBA melting temperature was only 21.7 °C, but T_m_ values for IPS1 and IPS2 were higher by 19.6 and 30 °C, respectively. These results confirm conclusions from CD spectra results that TBA and IPS analogues form different G4 topologies. The difference is especially evident for sodium-stabilized G-quadruplexes, where TBA forms unstable G4 structure at room temperature.

**Figure 2:**
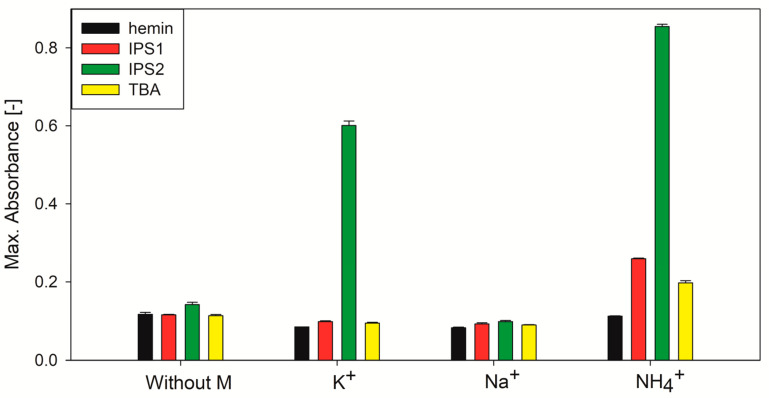
Catalytic activity of DNAzymes in the reaction of ABTS oxidation. Conditions: 10 mM Tris-HCl (pH = 8.0), 100 mM KCl, NaCl or NH_4_Cl, 0.0016% Triton X-100, 1 µM DNA, 1 µM hemin, 1 mM ABTS, 1 mM H_2_O_2_.

The activity of the DNAzymes based on the studied oligonucleotides was examined using ABTS as a substrate of peroxidase reaction and results are shown in Figure 2. As mentioned earlier, TBA typically does not possess peroxidase activity [31]. In the absence of coordinated cations all oligonucleotides exhibit only residual peroxidase activity comparable to that for hemin itself. TBA showed slightly higher activity only in the presence of an ammonium ion. As in the case of TBA, the DNAzyme based on the IPS1 showed higher activity only if NH_4_^+^ cations were present. On the contrary, the IPS2 DNAzyme showed remarkable high activity in the presence of both potassium or ammonium ions. Interestingly, low activity was observed in the environment of sodium ions, despite that both IPS1 and IPS2 formed more thermally stable G-quadruplexes in the presence of sodium cations. In 2011 Zhang et al. reported that the binding of thrombin by TBA enhanced the peroxidase activity of such a DNAzyme system [31]. We tried to reproduce those results but without success (Figure S3). 

The activity results shed more light on the structure formed by the IPS2 oligonucleotide. Activity of the DNAzyme depends on the topology of the G-quadruplex. When G-quadruplexes have loops protruding above the G-tetrad, they form steric hindrance for the end-stacking binding of hemin. This is the reason why parallel G-quadruplexes with all propeller loops (e.g., PS2.M or Cat G4) have the highest activity [26,27]. In typical parallel G-quadruplexes all loops are propeller and do not interfere with hemin binding. Since the IPS2 exhibited very high activity in comparison to TBA and IPS1, a substantial difference in G-quadruplex topology for IPS2 should exist. Recently, Esposito and co-workers reported similar TBA analogue with three IPS but at different locations in the strand [22]. Suggested topology included a 3 + 1 structure with one propeller loop. In the case of IPS2, very high peroxidase activity of DNAzyme and its CD signature confirmed the plausibility of an antiparallel structure with all propeller loop arrangements (Scheme 1E). The low activity of the IPS1 at potassium ion and IPS1 and IPS2 at sodium ion, as well as comparable CD spectra, suggest a similar topology of G4 for these systems. We propose the formation of a 3 + 1 structure C (Scheme 1) for IPS1 in sodium and potassium ions and a similar 3 + 1 structure D for IPS2 at sodium ions. For both 3 + 1 G4 (C and D), an enhancement in the peroxidase activity should be negligible and comparable to that for TBA, since lateral loops are expected to disturb in the binding of hemin. Experimental results (Figure 2) confirmed the above conclusion. Only structures with propeller loops and exposed terminal tetrads, shown in Scheme 1E, explains the increase in the activity of the IPS2 DNAzyme.

**Figure 3:**
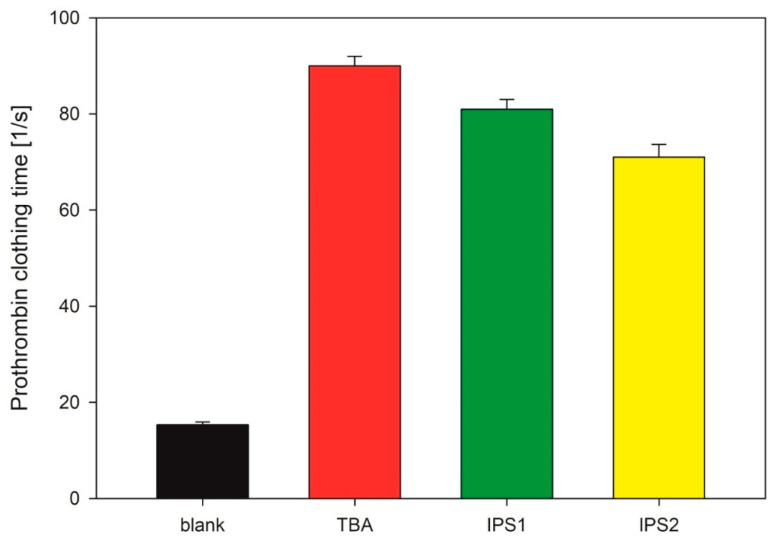
Fibrinogen clothing time measured in the presence of thrombin aptamers. Conditions: 10 mM PBS, 2 nM thrombin, 20 nM DNA, 1 µM fibrinogen.

Fibrinogen is a large, complex glycoprotein (340 kDa) consisting of pairs of three polypeptide chains named Aα, Bβ, and γ chains. Fibrinogen plays a central role in the mechanism of coagulation and thrombosis as an adhesion protein essential to platelet aggregation and as a precursor of insoluble fibrin that forms the definitive fibrin clot [33]. To examine the effect of the introduction of IPS in a TBA aptamer on the inhibition effect on thrombin activity, the fibrinogen clotting time was determined (Figure 3, Figure S4). The experiments showed that both IPS1 and IPS2 have slightly lower anticoagulant activity than TBA. Still, fibrinogen clotting time is much higher than for control experiment without any inhibitors. This means that the alteration of TT loops by both backbone structures and their spatial arrangement in G4 exerted minor effects on the anticoagulant properties of modified aptamers, even that TT loops are regarded as crucial structural elements of TBA responsible for thrombin binding.** The advantage of modified TBA that includes chemical modifications is expected resistance to degradation in biological samples. It was already evidenced that aptamers with inversion of polarity sites were resistant to nucleases up to 24 h, while TBA was degraded in 1 h [20]. Higher resistance to nucleases in biological samples is advantageous for further therapeutic applications.

## 3. Materials and Methods

### 3.1. Materials and Instruments

The DNA oligonucleotides (HPLC purified) were purchased from Eurogentec. (Belgium). The sequences of used oligonucleotides are gathered in Table 2. All oligonucleotides were used without further purification. The concentration of the oligonucleotides was quantified using UV-Vis spectroscopy at 85 °C with the following extinction coefficients at 260 nm (M^−1^, cm^−1^) T = 8700, G = 11,500. The hemin, 2,2′-azinobis(3-ethylbenzothiozoline)-6-sulfonic acid (ABTS), H_2_O_2_, and Triton X-100 were purchased from Sigma-Aldrich (Poznan, Poland). The hemin was dissolved in DMSO and the 1 × 10^−2^ M stock solution was stored in the freezer. Other reagents were of analytical grade and used as received. The absorption spectra and melting profiles were recorded using a Cary 100 UV-Vis Spectrophotometer (Agilent Technologies, Santa Clara, CA, USA). For the measurements of the DNAzymes activity, a M200 microplate reader (Tecan, Mannedorf, Switzerland) was used. The CD spectra were recorded using a J-810 spectropolarimeter (Jasco, Tokyo, Japan). All experiments besides melting profiles were carried out at 25 °C.

### 3.2. CD Spectroscopy and Melting Profiles Determination

The CD spectra were measured with 100 nm/min scanning speed and bandwidth of 1 nm. Spectra were recorded in quartz cells of 1 cm path length and averaged from 3 scans. Melting profiles were determined by the measurement of absorbance change at 295 nm with the change of temperature (15–90 °C). All samples contained 2 µM oligonucleotide, 10 mM Tris-HCl buffer pH = 8.0 and 100 mM required cation. Prior to the experiment the samples were denatured at 95 °C for 5 min and then cooled on ice for 15 min and left to cool down to room temperature. All measurements were conducted in 3 replicates.

### 3.3. DNAzyme Activity Measurement

The measurements were conducted using samples containing 1 µM oligonucleotide, 10 mM Tris-HCl buffer pH = 8.0, 0.0016 % Triton X-100, 1 µM hemin and 100 mM adequate salt (KCl, NaCl or NH_4_Cl). First, the samples were denatured at 95 °C for 5 min and then cooled on ice for 15 min. After the addition of colorimetric substrate ABTS (1 mM), samples were transferred into a microplate and the reaction was initiated by an addition of H_2_O_2_ (1 mM). The absorbance at 417 nm was monitored in 10 s intervals for 15 min. All measurements were conducted in 3 replicates.

### 3.4. Clothing Time Determination

The experiments were performed by recording the change of absorbance at 380 nm in time. Twenty nM oligonucleotides with 2 mg/mL fibrinogen in 1 mL of PBS were incubated for 5 min at 37 °C. Next, 100 µl of thrombin (1U) was added and the absorbance at 380 nm was measured for 10 min. The fibrinogen clothing time was calculated using a second derivative of A_380_ on a time curve. All measurements were conducted in 3 replicates.

## 4. Conclusions

Two IPS-modified analogues of thrombin-binding aptamer were designed and investigated. Both oligonucleotides, IPS1 (one inversion of polarity site) and IPS2 (three inversions of polarity sites) exhibited CD signatures typical for a chair-type G4 topology and were characterized by a higher thermal stability than TBA. The introduction of three inversions of polarity sites into the TBA molecule enhanced the peroxidase activity of the IPS2 DNAzyme, contrary to that of the IPS1 with one inversion site. These differences were attributed to differences in the G-quadruplex topologies of both molecules. The IPS1 (one inversion of polarity site) formed an antiparallel 3 + 1 structure with one propeller and two lateral loops [17], whereas the IPS2 (three inversions of polarity sites) also adopted an antiparallel G4 structure but with all propeller loops. This topology facilitates the binding of hemin and hence, explains the high activity of the IPS2. Our experiments proved that the introduction of inversion of polarity sites can drastically enhance the peroxidase activity of DNAzyme. These results also shed more light on possible structures adopted by TBA analogues. An unequivocal determination of an IPS2 structure would require more insightful studies with such techniques like NMR. We are planning to perform NMR experiments in the near future. Further, we examined the inhibitory effect of IPS-modified oligonucleotides on the fibrinogen clothing times. Both the IPS1 and IPS2 exhibited inhibitory effects on the thrombin activity. Despite the fact that the inhibitory effect was lower than that for the TBA oligonucleotide, the IPS1 and IPS2 possessed modifications that could make them more resistant to nucleases activity.

## Figures and Tables

**Table 1:** Melting temperatures calculated for studied oligonucleotides.

Name	Tm [°C], 100 mM K^+^	Tm [°C], 100 mM Na^+^
TBA	51.2 ± 1.1	21.7 ± 1.0
IPS1	54.5 ± 2.1	41.3 ± 0.5
IPS2	58.2 ± 2.5	51.7 ±1.8

**Table 2:** Sequences of TBA and its IPS modified oligonucleotides.

Name	Sequence
TBA	5′-GGT TGG TGT GGT TGG-3′
IPS1	3′-GGT-5′ -5′-TGG TGT GGT TGG-3′
IPS2	3′-GGT-5′ -5′-TGG TG-3′-3′-T GGT-5′-5′- TGG-3′

## Data Availability

The data presented in this study are available in this article and Supplementary Material.

## Supplementary Materials

The following are available online at https://www.mdpi.com/article/10.3390/ijms22157902/s1.
