# Supplementary material for: Thrombin-Binding Aptamer with Inversion of Polarity Sites (IPS): Effect on DNAzyme Activity and Anticoagulant Properties
Source: Int J Mol Sci. 2021 Jul 23;22(15):7902. doi: 10.3390/ijms22157902 (PMC8347255; doi:10.3390/ijms22157902)
Supplement: Supplementary file 1 [file ijms-22-07902-s001.zip › ijms-1289523-supplementary.pdf]

International Journal of
Molecular Sciences

MDPI

Thrombin-binding aptamer with inversion of polarity sites (IPS): effect on DNAzyme activity and anticoagulant properties

Joanna Kosman and Bernard Juskowiak

Department of Bioanalytical Chemistry, Faculty of Chemistry, Adam Mickiewicz University, 89b, 61-614 Poznan, Poland, kosman@amu.edu.pl, tel. +48 618291768

Supplementary materials

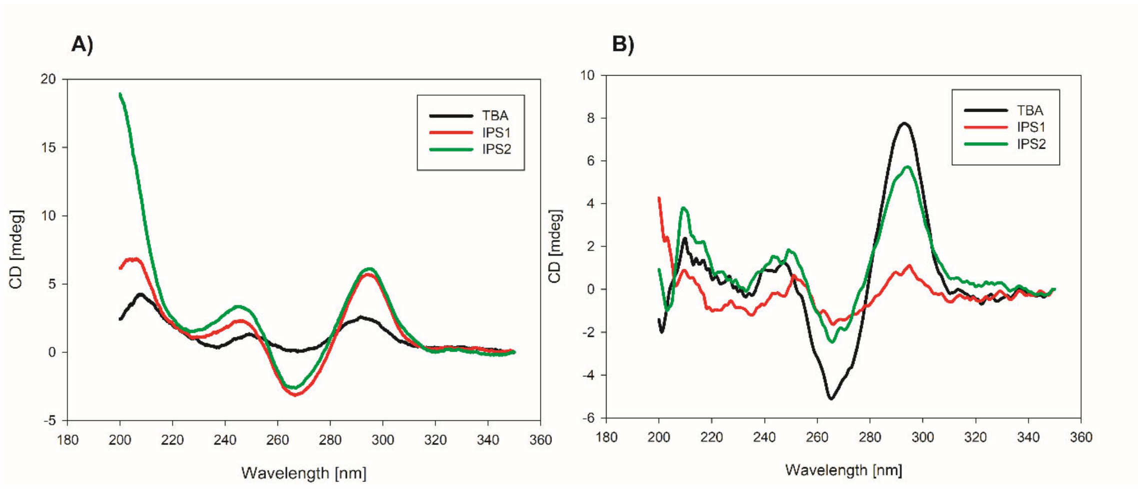

Figure S1. CD spectra of studied oligonucleotides in the presence of Na^+ (**A**) and NH_4^+ (**B**). Conditions: 10 mM Tris-HCl (pH = 8.0), 100 mM salt (NaCl or NH_4Cl), 2 μM DNA.

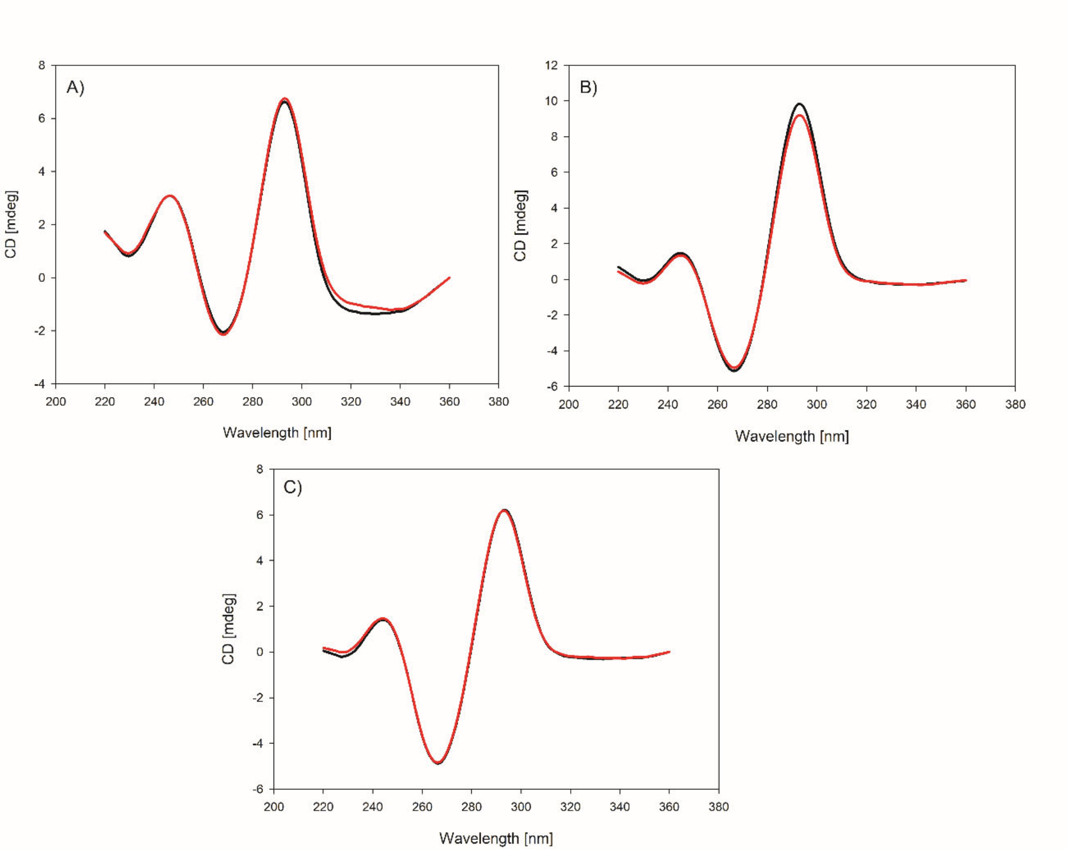

Figure S2. CD spectra of studied oligonucleotides prepared by slow (black) or fast cooling (red) for TBA (**A**), IPS1 (**B**) and IPS2 (**C**). Conditions: 10 mM Tris-HCl (pH = 8.0), 100 mM KCl, 2 μM DNA.

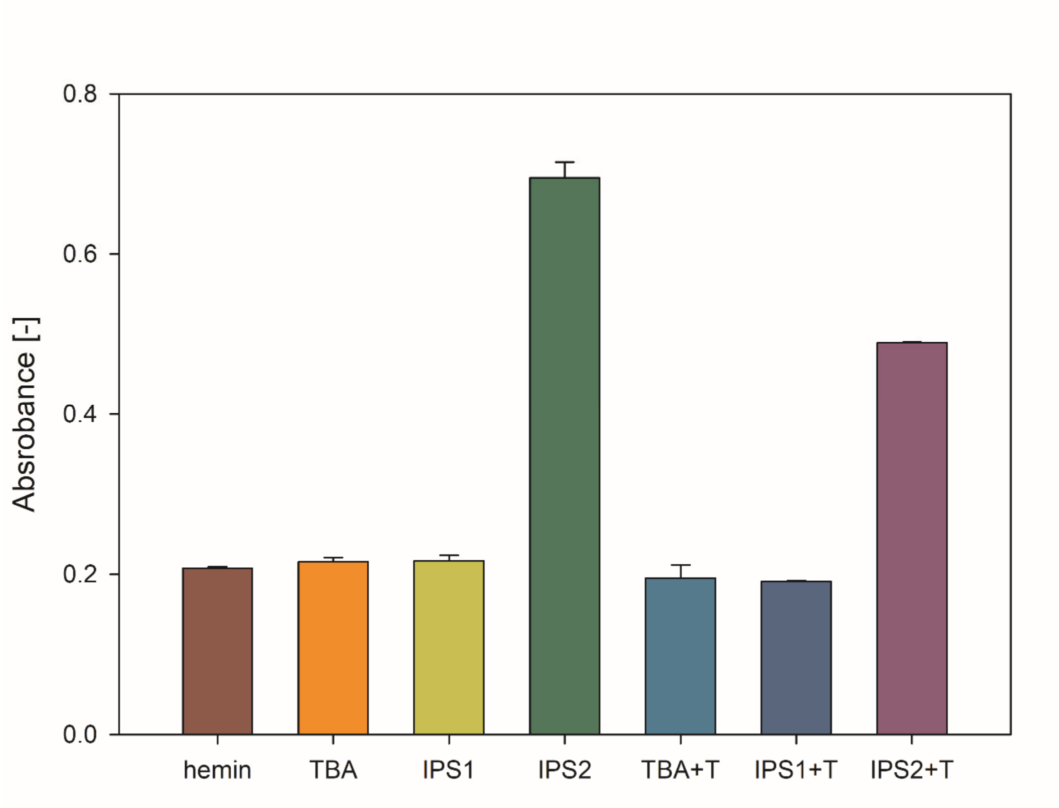

Figure S3. Effect of thrombin on catalytic activity of DNAzymes in the reaction of ABTS oxidation by hydrogen peroxide. Conditions: 10 mM Tris-HCl (pH=8.0), 100 mM KCl, NaCl or NH_4Cl, 0,0016% Triton X-100, 1 μM DNA, 1 μM hemin, 10 nM thrombin, 1 mM ABTS, 1 mM H_2O_2.

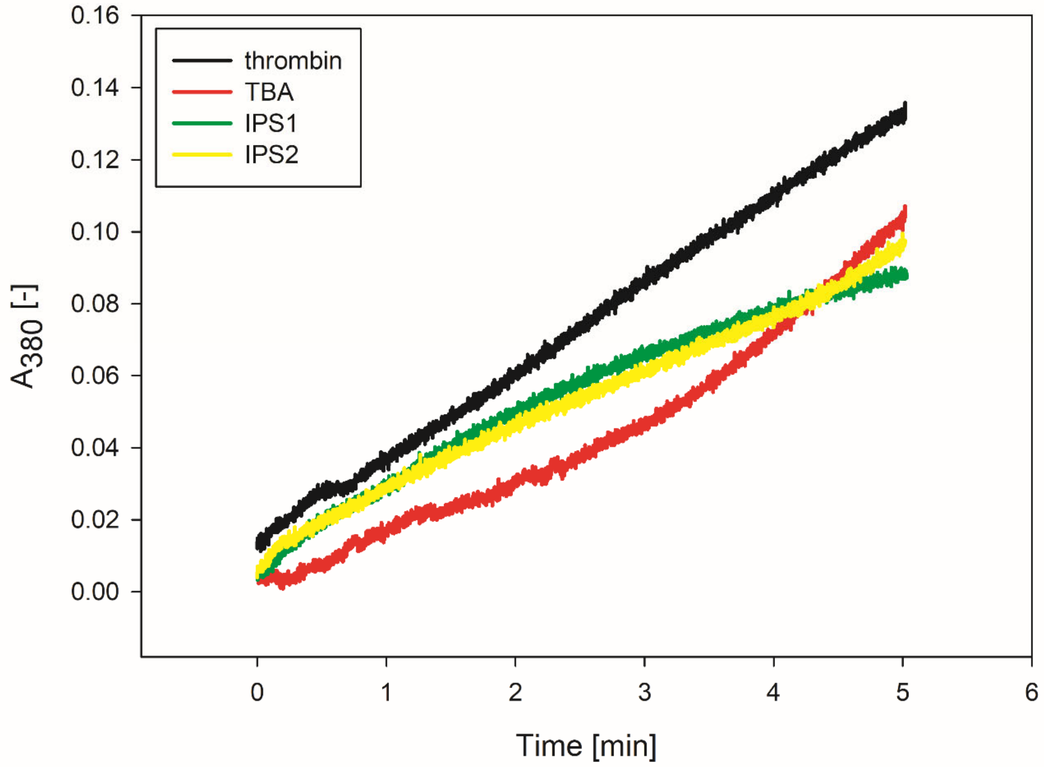

Figure S4. Change of absorbance at 480 nm with time in the reaction of fibrinogen to fibrin catalyzed by thrombin and inhibition effect of TBA, IPS1 and IPS2 oligonucleotides.
